# Projected hourly and regional energy demand for power heat and transport in New Zealand to 2050

**DOI:** 10.1038/s41597-025-06511-6

**Published:** 2026-01-12

**Authors:** Rafaella Canessa, Juan Carlos Osorio-Aravena, Rebecca Peer, Hans Christian Gils, Manuel Wetzel, Ashish Gulagi, Christian Breyer, Jannik Haas

**Affiliations:** 1 Sustainable Energy Research Group (SERG), Department of Civil and Environmental Engineering, University of Canterbury, Private Bag 4800, Christchurch, 8140 New Zealand; 2 Energy Center, Faculty of Physical and Mathematical Sciences, University of Chile, Santiago, Chile; 3 Institute for Regional Development, University of Granada, Granada, Spain; 4 German Aerospace Center (DLR), Institute of Networked Energy Systems, Curiestr. 4, 70563 Stuttgart, Germany; 5 LUT University, Yliopistonkatu 34, Lappeenranta, Finland

**Keywords:** Energy modelling, Renewable energy

## Abstract

Projections of energy demand are an essential dataset for analysing transition pathways and long-term investment planning. In New Zealand, however, publicly available datasets remain limited in scope and transparency. Existing studies have focused on electricity, while heat and transport have been only partially addressed. This dataset provides projections of final energy demand for New Zealand to 2050, at five-year intervals. Demand is disaggregated by sector (heat, transport, power), energy carrier (e.g., electricity, gas, liquid fuels), and technology (e.g., vehicles, boilers, heat pumps), and resolved to regional and hourly levels. Five exploratory scenarios capture alternative pathways of electrification, substitution of fossil fuels with biofuels and e-fuels, and behavioural change through shifts in transport modes and the adoption of diverse heating technologies across residential, commercial, and industrial applications. Projections were generated using a hybrid top-down/bottom-up approach informed by official statistics, policy targets, demographic and technical indicators. The dataset is designed for integration into energy system modelling and infrastructure planning, and its structure can be adapted for use in other national contexts.

## Background & Summary

Structured energy demand datasets are a foundation for evaluating climate mitigation scenarios, particularly in the context of national energy planning for the transition away from fossil fuels. Energy systems are a major contributor to global greenhouse gas emissions^1^ and projections of future demand are central to energy system modelling. Because energy demand depends on economic growth, demographics, evolving technologies, behaviours, and policies, such projections carry uncertainty. An increasing number of studies have developed demand projections to support national energy transitions in countries including Germany^2,3^, the UK^4,5^, Australia^6^, Japan^7^, Ethiopia^8^ and China^9^. Other studies have developed global datasets that are downscaled to the national level^10^. These studies provide demand scenarios with varying technology adoption pathways. They are used to support multi-sector energy modelling and to explore the implications of different transition strategies. Despite international commitments to the Paris Agreement^11^, many national strategies remain vague and lack sectoral detail or transparent assumptions. The Long-Term Strategies Scenarios and Pathways (LTS-SP) database^12^ highlights this gap and demonstrates the value of standardised and disaggregated projections across sectors.

Several characteristics make New Zealand a relevant case study for structured energy demand projections. Its electricity system is largely renewable (with renewables supplying over 80% of generation^13^) and is not interconnected with neighbouring countries, requiring domestic solutions for balancing supply and demand. Prior research has emphasised its suitability as a testbed for developing modelling tools and techniques that integrate technical and behavioural aspects of the grid. At the same time, New Zealand ranks among the most geopolitically stable countries^14^, which positions it as a secure location for investment in new energy carriers and international fuel trade. Together, these conditions underscore the value of developing transparent and detailed demand scenarios for the country, with potential reuse in both domestic planning and comparative international analyses.

The country has adopted emissions reduction^15,16^ and sectoral transition targets, including for transport^17,18^ and for heat^19,20^, where heat demand includes both buildings and industry, and covers both heating and cooling unless otherwise specified. However, these targets are not consistently quantified and do not outline detailed pathways for fuel switching and technology adoption. To date, no publicly available dataset provides multi-energy demand projections for New Zealand with both regional and hourly resolution. Existing national studies^21,22^ have largely concentrated on electricity, while modelling of heat and transport demand has been partial, often limited to process heat and road transport. Where technological change and fuel-switching are acknowledged^23^, key scenario assumptions and results are typically reported only at national or island level, or for single cities, and often with annual, seasonal, or monthly resolution rather than nationwide subnational and hourly coverage. Other analyses focus on single sectors or specific urban areas like the city of Auckland^24^. At the international scale, global databases provide electricity^25^ demand data for New Zealand directly. For heating^26^, and transport^27^, however, international datasets usually rely on top-down approaches that aggregate New Zealand within larger Asia-Pacific regions or apply downscaling from regional groupings. Such approaches can overlook New Zealand’s regional structure and sectoral characteristics, including the high penetration of residential electric heating (56% of New Zealand households use heat pumps as their main source of heat^28^) and the predominance of private vehicles in New Zealand’s transport sector (most travel occurs in fossil-fuelled light vehicles, while public transport, walking and cycling remain underutilised^15^). An OECD analysis^24^ specific to New Zealand has called for improved modelling capacity, spatial data, and long-term planning frameworks to support the energy transition and local decision-making.

This dataset^29^ contains projections of New Zealand’s final energy demand from 2020 to 2050, reported at five-year intervals. Demand is disaggregated by sector, fuel, and technology, and resolved to hourly and 16-region values. Five exploratory scenarios represent alternative combinations of technology adoption and fuel switching. Demand projections were generated with a hybrid top-down/bottom-up approach, and the disaggregation incorporated demographic and technical indicators.

The dataset^29^ is structured for reuse in a wide range of applications. Energy system modellers can integrate the projections into transition studies at national or regional scales. Policymakers and planners may use them to explore the implications of alternative technology pathways for infrastructure development, demand management, and sectoral transition strategies. The hourly profiles enable analysis of load dynamics and operational flexibility, while the regional detail supports assessments that capture geographic variation in energy demand. The scenario framework allows for sensitivity studies across contrasting futures, and the underlying methodological approach can be adapted to construct comparable datasets for other countries. By offering an open, structured and scenario-based resource, this dataset^29^ provides sectoral, spatial and temporal detail that can be applied in national planning and in international comparative analyses.

## Methods

The dataset^29^ was constructed by combining national energy statistics, policy targets, and efficiencies reported in the literature to model future energy demand. Projections are based on both local data and global modelling assumptions, and they are structured by scenario and sector. Regional and hourly demand resolution was achieved by combining sector-specific indicators with empirical profiles: demographic indicators and heat database proxies for the heat sector, transport activity statistics and charging profiles for the transport sector, and node-to-region mapping using wholesale electricity demand data for the power sector.

### Input data

This study uses a range of data sources:

- New Zealand datasets: Delivered energy data from the “Energy End Use Database (EEUD)”^30^; transport fleet statistics from the Ministry of Transport^31,32^; population projections, household numbers, and regional estimates from Stats NZ^33,34^; nodal electricity demand from the Electricity Authority (EA)^35–38^; and electricity consumption profiles for industrial, residential, and commercial sectors^39^.
- New Zealand studies and policy documents: National studies like “Whakamana i Te Mauri Hiko – Empowering Our Energy Future”^23^, two versions of the “Electricity Demand and Generation Scenarios (EDGS)”^21,22^ by the Ministry of Business, Innovation and Employment (MBIE), and the “Measuring emissions: A guide for organisations: 2024 detailed guide”^40^. Policy and target documents include the First and Second Emissions Reduction Plans from the Ministry for the Environment^15,16^. Sector-specific sources include:
  - Heat: “Gas Transition Plan Issues Paper”^41^; “Phasing out fossil fuels in process heat consultation document”^42^; “National Policy Statement for Greenhouse Gas Emissions from Industrial Process Heat 2023”^43^; chapter 15 of “Ināia tonu nei: A low emissions future for Aotearoa”^41,42,44^. and submissions and recommendations for industrial GHG emissions policy^45^.
  - Transport: “Liquid Biofuels Insights”^46,47^; “Green Freight: Strategic Working Paper”^48^; and “Domestic Transport Costs and Charges (DTCC) Study”^49^.
  - Power: Interim Climate Change Committee reports^50,51^.
- International datasets: technology efficiencies from the Danish Energy Agency, including individual heating efficiencies^52^ and industrial process heat efficiencies^53^.
- International studies: transport projections and evolving motor efficiencies^27^; hourly heating profiles^26^; global heating trends^10^; electricity projections and profiles^25^; and cross-validation of demand magnitudes^54^.

All primary datasets are government-released or public-domain sources, which permit reuse and redistribution for non-commercial purposes. These sources have been cited accordingly.

### Scenario definitions

The dataset^29^ includes five exploratory scenarios for projected energy demand from 2025 to 2050, at five-year intervals. These scenarios represent a range of possible pathways for heating and transport technology adoption in New Zealand. All scenarios share a common 2020 baseline and use the same calculation framework across sectors. Each scenario is compatible with New Zealand reaching net-zero emissions by 2050. The main differences lie in the pace of transformation in heat and transport sectors and in the mix of fuels that replace fossil demand. In all cases, any remaining liquid fuel use in 2050 is met by biofuels, e-fuels, or a combination of both.

E-fuels are included only where a residual portion of liquid-fuel demand persists, for example in hard-to-electrify applications (e.g., long-distance transport or high-temperature industrial process heat). Under New Zealand’s legislated 2050 net-zero target, any remaining liquid fuel demand must be met by sustainable non-fossil substitutes. E-fuels therefore serve as a boundary-condition option to ensure internal consistency with net-zero. Their deployment is modelled gradually and remains limited in volume, and their usage varies across scenarios. E-fuels are discussed in the decarbonisation literature^29,55,56^ due to improving conversion efficiencies, declining renewable-electricity costs, and their relevance where alternatives may be constrained.

The differences between the scenarios stem from varying assumptions about the pace and scale of electrification (direct and indirect), transport behaviour change in the form of modal shifts across passenger and freight modes (including road, rail, marine, and aviation), and the extent of fuel switching. A summary of the scenario differences is provided in Table 1.

- Global Projections (GP): Applies global scenario data^26,27,57,58^ to New Zealand’s 2020 baseline. Includes top-down assumptions about modal and technology shifts. It serves as a reference case and motivates the creation of locally tailored scenarios.
- National Targets (NT): Reflects current national energy and climate targets, including phase-outs of fossil heating technologies and increased electric vehicle (EV) uptake. Assumptions are based on published NZ policy documents and planning studies. Where national targets are not defined, assumptions follow emerging global trends.
- Electrification (ELEC+): Emphasises rapid and widespread direct and indirect electrification across heating and transport. Technology adoption is accelerated relative to NT, which is particularly notable for space heating (heat pumps) and passenger transport (EV).
- Biomass (BIO+): Focuses on expanded use of biomass and biofuels. Assumes favourable bioenergy policy and supply, with partial (higher than any other scenario) replacement of fossil fuels by solid, liquid, and gaseous biofuels.
- Hydrogen (H₂+): Explores the deployment of (green) hydrogen through e-fuels (indirect electrification) for hard-to-electrify applications. Includes significant substitution of fossil fuels with e-fuels in transport and process heat. In contrast, the other scenarios replace these same sectors with biomass (BIO+), increased electrification (ELEC+), or a mix including limited usage of e-fuels. Table 1 provides a complete comparison.

**Table 1:** Scenario definition summary and comparison.

Feature	GP (Global Projections)	NT (National Targets)	ELEC + (Electrification)	BIO + (Biomass)	H₂ + (Hydrogen)
Main driver	Global techno-economic models and projections.	New Zealand national emission targets and policies.	Strong policy or market preference for electrification.	Expanded role for bioenergy under favourable conditions.	Technology push or policy support for hydrogen.
Data source orientation	International studies.	New Zealand national targets, strategies and consultations.	NT baseline with accelerated and enhanced electrification assumptions.	NT baseline with enhanced biomass	NT baseline with enhanced hydrogen uptake.
Technology preference	Mixed, based on global technology shares.	Policy-guided technology transitions.	Optimistic direct electrification (e.g. EVs, heat pumps), some indirect (e-fuels).	Biomass fuels (solid, liquid, gas).	E-fuels derived from green hydrogen.
Fuel mix trajectory	Fossil fuel phase-out aligned with global studies.	Fossil fuel phase-out aligned with NZ targets.	Fossil fuel rapidly displaced by electricity.	Fossil fuels replaced by biomass-derived fuels.	Fossil fuels replaced by hydrogen-derived fuels.
Assumed efficiency gains	Global motor and heating efficiencies.	Moderate efficiency gains from electrification and fuel switching.	High, based on electric motor and heat pump efficiency.	Medium, varies by biofuel type and application.	Medium, including conversion losses from hydrogen synthesis.
Electrification role	Present (directly and indirectly for H₂ and e-fuel production).	Moderate (aligned with targets).	High direct use (core transition pathway), indirect use for H₂ and e-fuel production.	Present (directly and indirectly for H₂ and e-fuel production).	Present (directly and indirectly for H₂ and e-fuel production).
Hydrogen role	Limited, present for non-electrifiable loads.	Limited, present for non-electrifiable loads.	Limited, present for non-electrifiable loads.	Limited, mostly displaced by bio-alternatives.	Present, focused on hard-to-electrify sectors.
Biomass role	Limited.	Limited, blended to meet some policy gaps.	Limited.	Present, focused on hard-to-electrify sectors.	Limited.
Use case / scenario purpose	Illustrates consequences of non-localised assumptions.	Reference based on national commitments and suggestions.	Explores impact of accelerated electrification	Explores impact of increased biomass use.	Explores impact of increased hydrogen-based alternatives.

These scenarios do not intend to predict or prescribe the future. Instead, they explore how energy demand could evolve under different conditions, to support the exploration of alternative futures and informing long-term planning.

### Sectoral approach

The demand projection framework applies a hybrid approach between a granular bottom-up assessment (for the power sector) and a top-down approach (for heat and transport), based on data availability and sector characteristics. For the baseline diagnostic, delivered energy was obtained from the EEUD^30^. For heat, the useful energy was then obtained using technology efficiencies from the Danish Energy Agency (DEA)^52,53^. For transport, passenger-kilometre (p-km) and tonne-km (t-km) metrics were calculated based on literature conversions^27^ and fleet statistics^31,32^. For power, nodal electricity consumption was sourced from the EA^39^, and aggregated according to regional boundaries.

### Heat sector

Heat demand projections were developed by converting delivered energy from national datasets into useful energy using technology-specific efficiency factors. This “useful energy” baseline was then projected under the five scenarios, with future delivered energy calculated based on the evolving efficiencies of the chosen heating technologies.

#### Baseline diagnostic: national heat energy demand

National pre-COVID-19 heat demand was estimated using the EEUD^30,59^, which reports delivered energy by sector and end use. Delivered energy values were adjusted using technology-specific efficiency factors to estimate useful heat demand. Although 2019 was warmer than the long-term baseline, reports indicate near-average annual temperatures in several regions and no prolonged extreme event^60^, and national heating and cooling demand were within normal interannual variability^29^.

The EEUD sectoral breakdown (agriculture/forestry/fishing, commercial, industrial, and residential) was maintained. Fuels were grouped into broader categories (e.g., gas includes natural gas, LNG, and biogas; liquid fuels include diesel, fuel oil, and petrol).

Efficiency values were primarily sourced from the DEA technology catalogues for industrial process heat^53^ and heating installations^52^. Gas cooking efficiency was sourced separately^61^. The baseline diagnostic is provided in Sheet H1_Diagnostic.

#### Projection of useful heat demand

Useful heat demand was projected based on a global-local study^26^ that provides annual heat demand estimates for several countries by category. Heating applications from the EEUD^30^ sectors (agriculture/forestry/fishing, commercial, industrial, residential) were grouped into the categories used in literature^26^: Industrial Heat Demand (IHD), Space Heating Demand (SHD), and Hot Water Demand (HWD). A fourth category, Cooking Heat Demand (CHD), was added. We aligned sectoral end uses with temperature levels: low-temperature heat below 100 °C (hot water, space heating, low-temperature processes), medium-temperature heat between 100 and 300 °C (cooking and medium-temperature processes), and high-temperature heat above 300 °C (high-temperature processes). Cooling demand, including space cooling and refrigeration, was also considered. Keiner *et al*.^26^ project IHD, SHD, and HWD based on drivers such as population, climate, and economic activity. Their year-to-year variations were applied to New Zealand data, with CHD following the HWD curve. Projections of useful heat demand were provided in Sheet H2_Projections.

#### Projection of scenario-based technology adoption and national heat energy demand to 2050

Different technology adoption curves were defined for each end use within each category. For instance, in the largest demand category, high-temperature heat for IHD, the technologies considered include boilers and direct-fired heating systems. Fuels considered include gas, coal, liquid fuels, and electricity, with their shares varying depending on the scenario.

For each heat end-use category (i.e., IHD, SHD, HWD and CHD), distinct technology adoption curves to 2050 were defined under the five scenarios. Each curve reflects shifts in technology shares (e.g., heat pump, gas boilers, direct-fired systems, etc.) and associated fuels (gas, liquid fuels, electricity, coal, biomass). The fuel composition was adjusted over time to reflect replacement dynamics, with varying shares of fossil fuels, e-fuels and biofuels. A summary of the assumptions is shown in Table 2.

**Table 2:** Summary of scenario assumptions for the heat sector.

				Scenario				
Mode	Application	Type	Energy demand 2019 (TWh)	GP (Global Projections)	NT (National Targets)	ELEC + (Electrification)	BIO + (Biomass)	H₂ + (Hydrogen)
Heating	Industrial heat	High temp. (>300 °C)	17.3	Gas share remains prominent. The remaining is electrified	Gas phase-out by 2030^41^. Suggested switch to biogas or heat pumps^19^, follows GP.	Mostly directly electrified in 2050. Some indirect electrification.	Higher share of biogas demand in gas composition.	Gas usage remains constant with increasing relevance of e-gas.
		Mid temp. (100–300 °C)	14.0	Gas share remains constant. Increase in electricity and biomass.	Gas phase-out by 2050^41^ and existing low- and medium-temperature coal boilers must be phased out by 2037^41,42,44^. Maintains geothermal share constant.	Geothermal share remains constant, and rest is electrified.	Biogas has a larger relevance and increased role for direct biomass use.	Increasing role of e-gas in gas demand composition.
		Low temp. (<100 °C)	3.5	Electrification leads but some gas remains in the mix.	Gas phase-out by 2030^41^ and existing low- and medium-temperature coal boilers must be phased out by 2037^41,42,44^.	As NT, accelerated.	Biofuel share in liquid fuel demand is increased.	As NT, with increased use of e-fuels in liquids and e-gas within the gas mix.
	Space heating	Low temp. (<100 °C)	11.7	Mostly electrified, the rest is biomass.	Phase out gas in buildings by 2030^16^, fully electrified by 2050.	As NT, accelerated.	Increased biomass role, follows GP.	
	Hot water	Low temp. ( < 100 °C)	11.0	Mostly electrified with uptake of solar and biomass.	Electrified with existing solar share constant.	As NT, accelerated.	Increased biomass role, follows GP.	
	Cooking	Mid temp. (100–300 °C)	2.3	Fully electrified.	Gas phase-out in buildings by 2030^16^, fully electrified by 2050.	As NT, accelerated.	Increased biomass role, follows GP.	
Cooling	Industrial cooling	Refrigeration	4.4	Fully electrified.				
	Space cooling	Cooling	1.1					

The previously projected useful heat demand was converted back to delivered (end-use) energy using the technology splits defined for each scenario. Efficiency assumptions from the technical literature^52,53^ were applied year by year to obtain end-use energy for heating and cooling. The full workflow, adoption curves and results are available in Sheet H3_Scenarios.

#### Regional split and hourly profile for heat energy demand

**Fig. 1:**
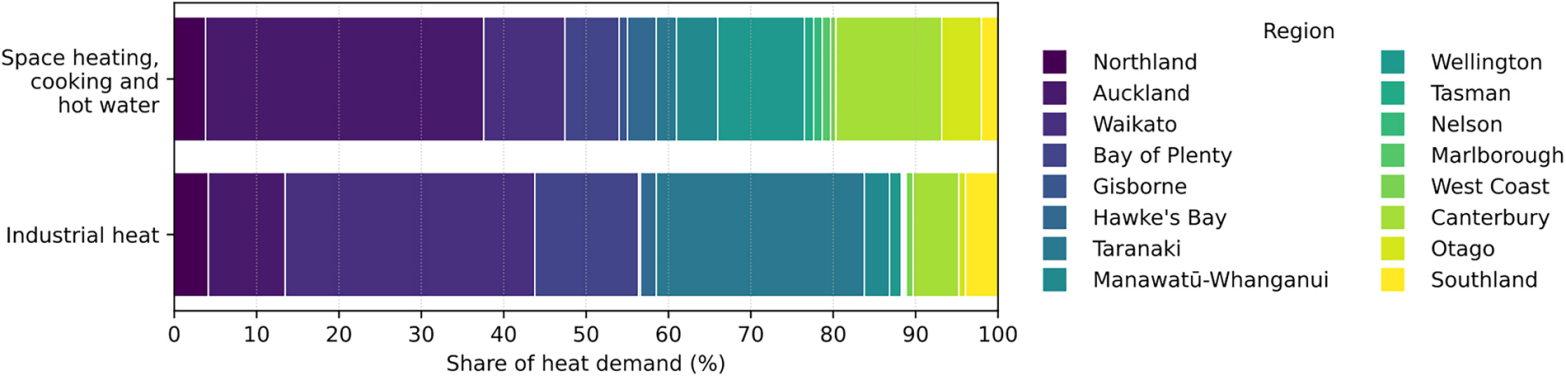
Distribution of heat demand across regions in New Zealand.

**Fig. 2:**
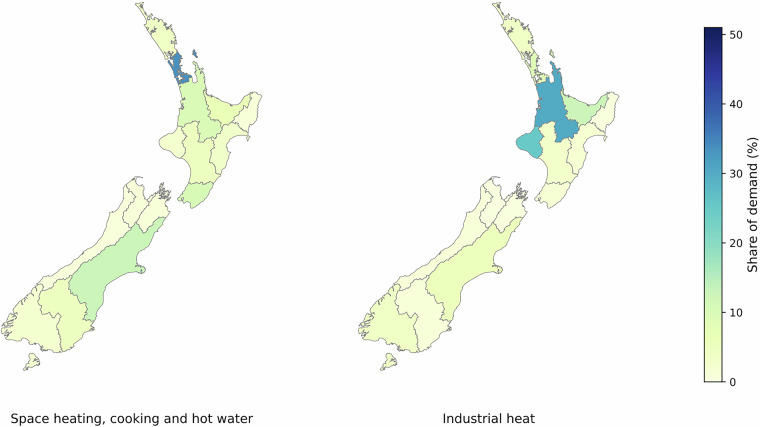
Distribution of heat demand across regions in New Zealand, map view.

National heat demand is split into 16 regions for each fuel and application. Figure 1 shows the regional distribution of heat demand by application and fuel. Figure 2 provides a map-based view of the same regional split. For IHD, regional fuel shares from the Regional Heat Demand Database^62^ were used as a proxy. For SHD, HWD and CHD, sub-national demographic data^33^ was applied.

Load profiles were built from yearly totals by scenario and fuel, combined with application shares (IHD, SHD, HWD, CHD). Hourly profiles^26^ were applied to SHD, HWD, and IHD; CHD adopts the HWD profile. Each regional profile was scaled so that the 8,760-hour sum matches the annual electricity demand for heat (GWh). Application profiles were then aggregated into a national heat profile. Hourly and regional demand data are provided in Sheet H4_Disaggregation.

### Transport sector

Transport energy demand was converted into passenger-kilometres (p-km) and tonne-kilometres (t-km) using energy intensity values, projected to 2050 under five scenarios, and then converted back to energy demand based on evolving technology performance.

#### Baseline diagnostic: national transport energy demand

Pre-COVID-19 transport energy demand for road, rail, marine, and aviation was obtained from the EEUD^30^ and converted to p-km and t-km^27^. Fleet composition by propulsion type (e.g. combustion, electric) across light-duty vehicles (LDV), two-/three-wheelers (2 W/3 W), buses and heavy-duty vehicles (HDV) was sourced from Ministry of Transport statistics^32^. These diagnostics are provided in Sheet T1_Diagnostic.

#### Projection of transport service

Transport service (p-km, t-km) was projected to 2050 using growth rates from national transport statistics (Table 145)^27^, scaled to reach 100%. Year-by-year variations were applied to baseline values, with results in Sheet T2_Service.

#### Scenario-based projection of transport activity, technology adoption and national transport energy demand to 2050

Each transport mode was assigned a set of propulsion technologies with adoption varying by scenario. LDVs include internal combustion engine (ICE), battery electric vehicles (BEV), plug-in hybrid electric vehicles (PHEV), and fuel cell electric vehicles (FCEV); rail uses electricity or liquid fuels; marine considers electricity, hydrogen, gas, and liquid fuels; and aviation includes electricity, hydrogen, and liquid fuels. Gas and liquid fuels were further disaggregated into fossil, biofuel, and e-fuel categories, and their shares evolve over time via fuel replacement curves. These trajectories reflect the gradual substitution of fossil fuels with renewable and synthetic alternatives, consistent with the heating sector approach.

Mode- and technology-specific energy demand (GWh) was derived using conversion rates (Tables 17-18)^27^. Calculations are provided in Sheet T3_Scenarios and a summary of the scenario differences is provided in Table 3.

**Table 3:** Summary of scenario assumptions for the transport sector.

				Scenario assumption summary				
Mode	Application	Type	Energy demand 2019 (TWh)	GP (Global Projections)	NT (National Targets)	ELEC + (Electrification)	BIO + (Biomass)	H₂ + (Hydrogen)
Passenger	Road	Light Duty Vehicle	38.9	Preference for direct electrification, small FCEV presence.	Reflects NZ fleet and emissions targets. High direct electrification, minimal liquid fuels by 2050.	Faster direct electrification than NT. Liquid fuels phased out by 2050.	Higher share of biomass-derived fuels (biofuels, biogas) than NT.	Higher share of hydrogen-derived fuels (e-fuels, e-gas) than NT.
		Bus	1.1					
		Two- and Three-Wheelers	0.2					
	Aviation Passenger		4.2	Liquid fuels present but fossils are replaced with e-fuels. Some electrification and direct hydrogen use.	Fully liquid fuel based (fossil fuel phase-out). No direct electrification or hydrogen.	Some direct electrification	Same as NT but with some liquid biofuel presence.	Same as GP, faster fuel switching to e-fuel.
	Rail Passenger		0.1	Fully electrified by 2050.				
Freight	Road	Heavy Duty Vehicle	13.1	Phase out of fossil fuels, some direct electrification and FCEV.	Rise of electrification with higher liquid fuels (e-fuels and biofuels) presence than GP.	Higher liquid fuels (e-fuels and biofuels) than NT.	Higher liquid fuels (e-fuels and biofuels) than NT.	Higher liquid fuels (e-fuels) than NT. Minimal presence of FCEV.
	Rail Freight		1.3	Fully electrified rapidly.	Fully electrified by 2050.	Fully electrified. Faster than NT.	Partially electrified. Liquid fuels are e-fuels and biofuels.	Partially electrified. Liquid fuels are mostly e-fuels.
	Marine Freight		1.0	Decline in liquid fuel use, presence of direct electricity and hydrogen use.	Mostly liquid fuel based with some gas and electrification.	As NT but faster electrification.	As NT but higher presence of biomass derived fuels.	Same as GP. Liquid and gas fuels are fully e-fuels/e-gas.

#### Regional split and hourly profile for transport energy demand

**Fig. 3:**
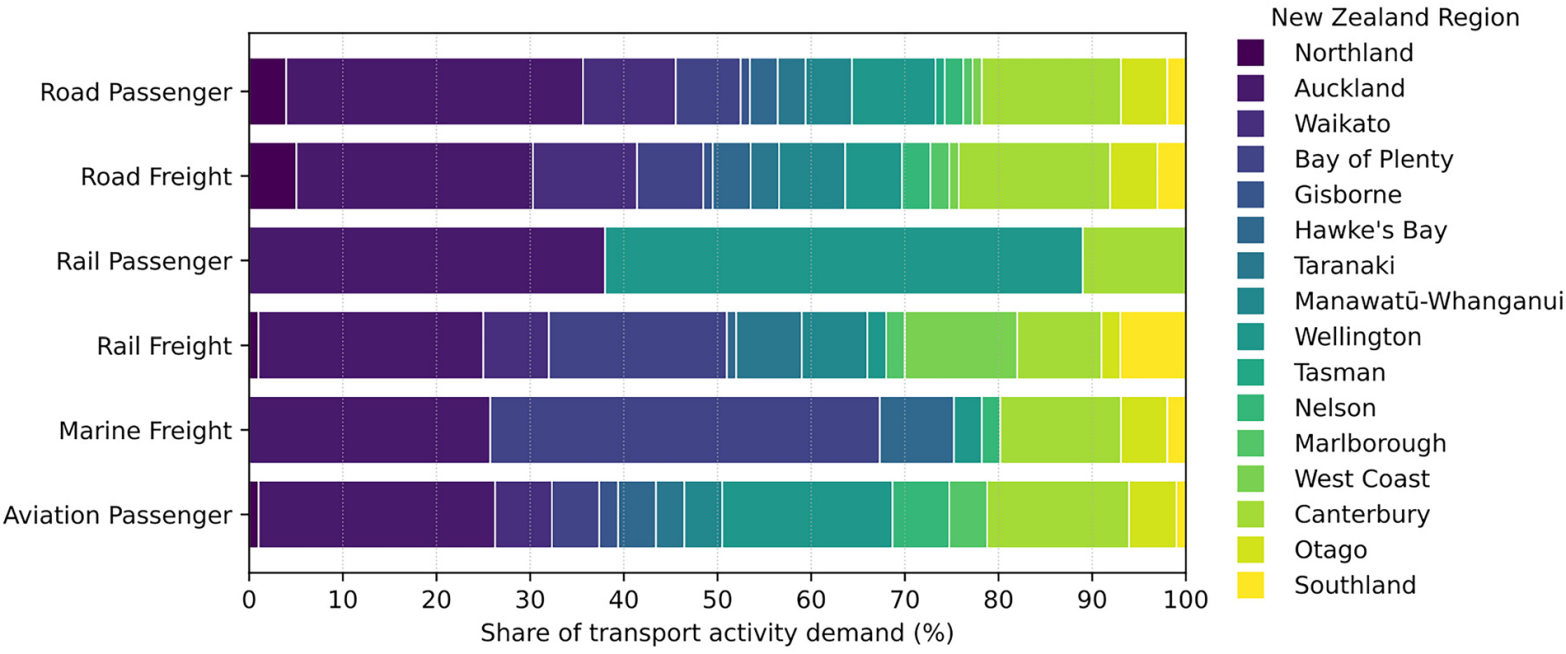
Distribution of transport activity demand across regions and transport modes in New Zealand.

**Fig. 4:**
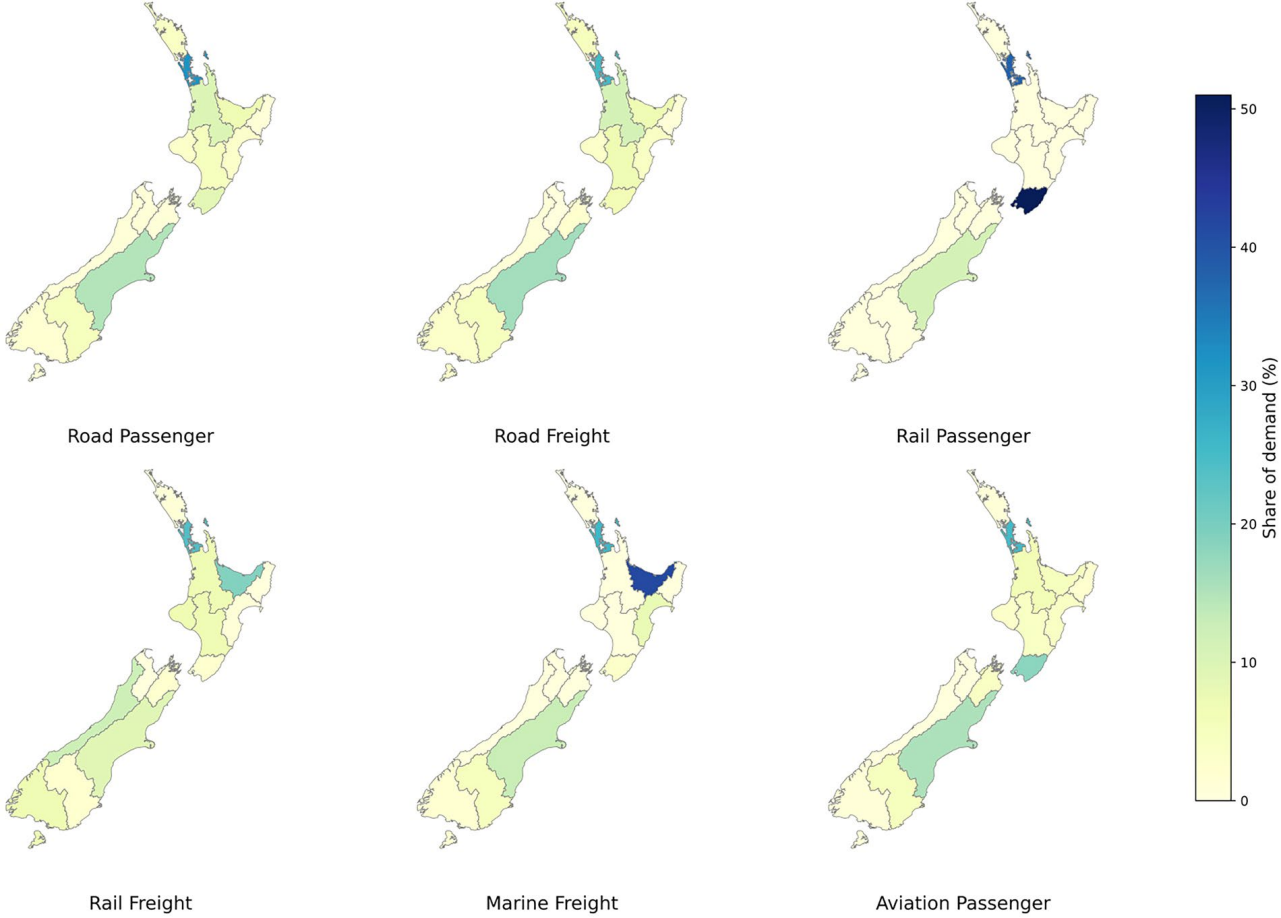
Distribution of transport activity demand across regions and transport modes in New Zealand, map view.

The regional distribution of road transport demand for LDV, 2 W/3 W and HDV is based on values calculated by the Ministry of Transport^49^. Bus p-km were taken from national statistics (Table C.8, Appendix C7)^49^, while rail passenger demand was derived from the p-km shares in the same table. Rail freight demand used the total t-km reported in national freight data^63^. Aviation passenger demand was allocated using flight information (Table C.5, Appendix C4)^49^. Marine freight volumes were taken from port-level cargo statistics^64^ and assigned to the corresponding regions. The distribution of transport activity demand across regions and modes is shown in Figs. 3, 4 provides a map-based view of the same regional split. The calculations for the regional allocation are provided in Sheet T4_Regions. Disaggregated values for each carrier and region are further organised in unstacked tables in Sheet T5_Carriers.

Hourly transport-electricity demand was constructed by weighting reference load profiles with scenario- and region-specific mode shares, then scaling to match each year’s total demand. LDVs followed representative charging profiles for the North and South Islands^65^, HDVs were assigned an overnight charging profile. Rail, marine and aviation electrification were assumed to follow flat load shapes. For each scenario, region and year, mode shares (road passenger ≈ 40–65%, road freight ≤45%, rail 1–55%, marine <15%, aviation <5%) were applied, normalised and linearly combined. The resulting 8,760-hour profiles sum to annual transport-electricity demand (GWh). Aggregated regional and hourly results are provided in Sheet T6_Elec_Profiles.

### Power sector

The power sector analysis excludes electricity used for heating and transport, as this demand is captured separately. Nodal wholesale electricity data from EMI^37^ and sectoral electricity consumption from the EEUD^30^ were used to estimate base electricity demand for the power sector. The electrification of heat and transport is addressed separately.

#### Baseline diagnostic: power sector energy demand (regional and hourly)

Electricity demand data for the “present day” (2019, pre-COVID) was sourced from EMI^37^. Half-hourly nodal data for each trading period were retrieved and aggregated to an hourly resolution per node. The data was accessed via the EMI platform under: Wholesale > Reports > Tag: All Tags > Demand Trends, using the following parameters:

- Date range: 1 January 2019 to 31 December 2019 (extracted monthly)
- Region type: Node
- Time scale: Trading period (half-hourly)
- Display units: GWh

Electricity consumption was split by sector (power, heat and transport) using the EEUD^30^. Since electrified transport accounts for less than 0.3% of total electricity use, only heat-related demand is excluded from the power sector demand profile at this stage. The adjustment was made by subtracting the electrified portion of the heat sector (IHD, SHD, CHD and HWD) for the base year.

#### Projection of national power sector energy demand to 2050

The electricity demand projection for the power sector is scaled according to relative variation in hourly load magnitudes and is based on New Zealand load projections from a global study^25^. This projection focuses solely on the direct power demand and explicitly excludes the additional electricity demand from other sectors in the energy system, like transport and heating.

#### Regional split and hourly profile for power sector energy demand

Regional grouping was done using a bottom-up approach, grouping nodes according to New Zealand’s 16 administrative regions. Information from wholesale electricity demand was geographically mapped using the NPS (Network Supply Points) data^36,38^ and Transpower site information (AC stations, DC stations, and Tees sites)^66^. The “TUI” point, which geographically falls into the “Bay of Plenty” region, was assigned to “Gisborne” in agreement with information from Transpower^67^ and the Gisborne District Council^68^.

### Data processing tools

Data processing and analysis were conducted using Microsoft Excel and Python (3.10.0). Python libraries employed include pandas (2.3.1) for data handling, numpy (2.2.6) for numerical operations, and matplotlib (3.10.3) for visualisation. Python standard libraries (e.g., os, sys, re, argparse, typing) were used for file handling and process management.

## Data Record

The dataset is available at Zenodo (10.5281/zenodo.17191686)^29^. It consists of four sectoral workbooks, a summary workbook, a supporting workbook, the hourly profile variants, and a small code example folder. All materials follow consistent field definitions:

- Scenario: Global Projections, National Targets, ELEC+, BIO+, H₂+.
- Year: 2020 to 2050 in 5-year increments.
- Region: New Zealand’s 16 administrative regions.
- Units: stated in each sheet. Primarily GWh (annual), GW (hourly), or % (shares).

The file Heat.xlsx contains data for the heat sector, including baseline diagnostics, projections of useful heat demand, scenario-based technology pathways, regional disaggregation, and validation outputs. The structure of this workbook is summarised Table 4.

**Table 4:** Overview of sheets and variables included in Heat.xlsx.

Sheet	Description	Row structure	Column structure	Units
H1_Diagnostic	Baseline delivered and useful energy	Fuel types	Heat applications and temperature levels	GWh
H2_Projection	Projections of useful heat demand	Application, temperature category, technology	Years 2020–2050	GWh
H3_Scenarios	Scenario-based adoption pathways	Application, temperature category, technology	Years 2020–2050	% of useful heat or GWh
H4_Disaggregation	Regional heat demand by carrier	Scenario, sector, carrier, region	Years 2020–2050	GWh
H5_Validation	Comparison with external benchmarks	Scenarios	Years 2020–2050	GWh or %

The file Transport.xlsx contains diagnostic transport activity, projected service demand, scenario-based technology pathways, regional allocation shares, hourly EV charging profiles, and validation tables. The structure is summarised in Table 5.

**Table 5:** Overview of sheets and variables included in Transport.xlsx.

Sheet	Description	Row structure	Column structure	Units
T1_Diagnostic	Baseline transport activity and energy	Transport modes	Activity, energy, intensity	p-km, t-km, TJ, kWh
T2_Service	Projected service demand and motor-type shares	Transport modes and motor-type groups	Years 2020–2050	p-km, t-km, %
T3_Scenarios	Scenario-based technology adoption	Mode, category, fuel	Years 2020–2050	GWh
T4_Regions	Regional allocation shares	Region, subsector, carrier	Years 2020–2050	%
T5_Carriers	Annual demand by carrier	Scenario, region, year, subsector, carrier	Carrier fields	GWh
T6_Elec_Profiles	Hourly EV charging demand	Scenario, region, year, sector, carrier	t0001–t8760	GWh (hourly)
T7_Validation	Comparison to external sources	Scenarios	Years 2020–2050	TWh

The file Power.xlsx contains electricity consumption diagnostics, projections of hourly power-sector demand, and validation against published studies. Its structure is summarised in Table 6.

**Table 6:** Overview of sheets and variables included in Power.xlsx.

Sheet	Description	Row structure	Column structure	Units
P1_Diagnostic	Current electricity consumption	Sector groups	Energy values	TJ, TWh, %
P2_Projection	Hourly electricity consumption (power sector)	Hours t0001–t8760	Node, year, sector, carrier	GWh per hour
P3_Validation	Comparison with external benchmarks	Scenarios vs external sources	Years 2020–2050	TWh or GW

The file Energy_summary.xlsx provides harmonised national and regional demand tables and hourly electricity profiles for all sectors. The structure is summarised in Table 7.

**Table 7:** Summary of national, regional, and hourly datasets in Energy_summary.xlsx.

Sheet	Description	Row structure	Column structure	Units
E1_National	Total national energy demand	Scenario, sector	Years 2020–2050	GWh
E2_Regional	Regional demand by scenario, sector, and fuel	Scenario, sector, fuel, region	Years 2020–2050	GWh
E3_Hourly_Heat	Hourly heat-sector electricity demand	Scenario, region, year, sector, carrier	t0001–t8760	GWh
E3_Hourly_Transport	Hourly transport-sector electricity demand	Scenario, region, year, sector, carrier	t0001–t8760	GWh
E3_Hourly_Power	Hourly electricity consumption within power sector	Scenario, region, year, sector, carrier	t0001–t8760	GWh

The file Supporting_data.xlsx contains supporting variables, including carbon-intensity reference data and sensitivity multipliers. Its structure is summarised in Table 8.

**Table 8:** Supporting values included in Supporting_data.xlsx (carbon factors and sensitivity multipliers).

Sheet	Description	Row structure	Column structure	Units
Carbon	Grid emission factors^40^, Emissions Trading System (ETS) auction prices^74^, IPCC price points^75^	Historical years or ETS months	Emission factors and auction-price fields	gCO₂/kWh, NZD/tCO₂, USD/tCO₂-eq
Multiplier	High/low multipliers for key model drivers	Sector, parameter type, driver	Base, low, high multipliers	Dimensionless

The file Heat_profile_with_variants.csv contains hourly electricity demand for the heat sector, including warmer-year and cooler-year variants created based on Heating Degree Day (HDD) and Cooling Degree Day (CDD) scaling. The file structure is shown in Table 9.

**Table 9:** Structure of the heat-sector hourly variant profiles (Heat_profile_with_variants.csv).

Structure	Description	Units
Variant, scenario, region, year, sector, carrier	Hourly heat-sector electricity demand for baseline, warmer-year, and cooler-year variants	GWh (t0001–t8760)

A small reproducibility package is included as code_example.zip. It contains a working script, a pinned environment file, and documentation. Its contents are summarised in Table 10.

**Table 10:** Components of the reproducibility example (Code_example folder).

File	Description
rebuild_example.py	Example script that loads regional and hourly data for a selected sector, scenario, and year, and reconstructs annual totals.
requirements.txt	Pinned package versions used by the example.
README.md	Step-by-step guide describing how the example relates to the published data.

### Contextual alignment with global scenario families

The scenarios in this dataset^29^ were not designed within the structure of global scenario families like the Shared Socioeconomic Pathways (SSPs)^69^ or their associated climate trajectories in the Representative Concentration Pathway (RCP)^70^. However, some users may find it useful to understand how the narrative features of our scenarios relate at a high level to these broader frameworks. Table 11: Descriptive mapping of scenario narratives to broadly comparable global pathway families. This mapping is intended to assist interpretation only and does not imply alignment with formal SSP-RCP scenarios. Table 11 therefore provides a qualitative, post-hoc mapping based on general similarities in underlying drivers (for example, population trends, heating-demand assumptions, or the pace of electrification).

**Table 11:** Descriptive mapping of scenario narratives to broadly comparable global pathway families.

Scenario	Key underlying drivers	Indicative SSP-RCP tendency
Global Projections	Useful heat demand based on Keiner *et al*.^26^; UN medium-fertility population; IPCC AR4 B1 temperature trend (moderate change); moderate efficiency improvements	~SSP2–RCP4.5 tendency
National Targets	Net-zero by 2050; moderate electrification acceleration; same demographic & climate inputs as GP	Between SSP2–4.5 and SSP1–2.6 tendencies
ELEC+	Strong electrification and efficiency improvements; reduced liquid-fuel demand	SSP1–2.6 tendency
BIO+	Higher availability of sustainable biomass; similar socioeconomic backdrop to GP	Within SSP2 range
H₂+	Hydrogen uptake in transport/industry; higher technology substitution	Between SSP1 and SSP2 tendencies

This mapping is intended to assist interpretation only and does not imply alignment with formal SSP-RCP scenarios.

This contextualisation does not imply consistency with SSP emissions budgets, climate outcomes, or modelling structures.

## Data Overview

**Fig. 5:**
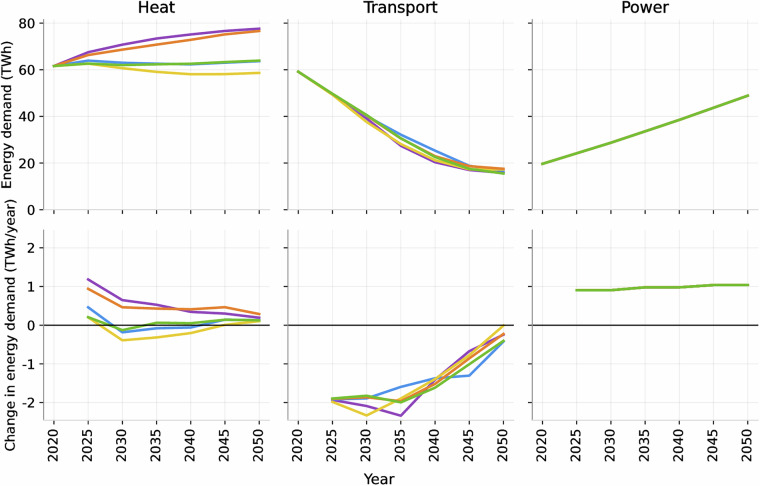
Sectoral energy demand trajectories (top row) and annual absolute change (bottom row). Results are shown for heat, transport, and power sectors across all five scenarios (Global Projections, National Targets, Electrification+, Biofuels+, and Hydrogen+). The top panels show national energy demand in TWh, while the bottom panels show the annual change in energy demand (TWh/year), computed as the difference between consecutive time points. A horizontal zero line is included to indicate transitions between increasing and decreasing demand.

**Fig. 6:**
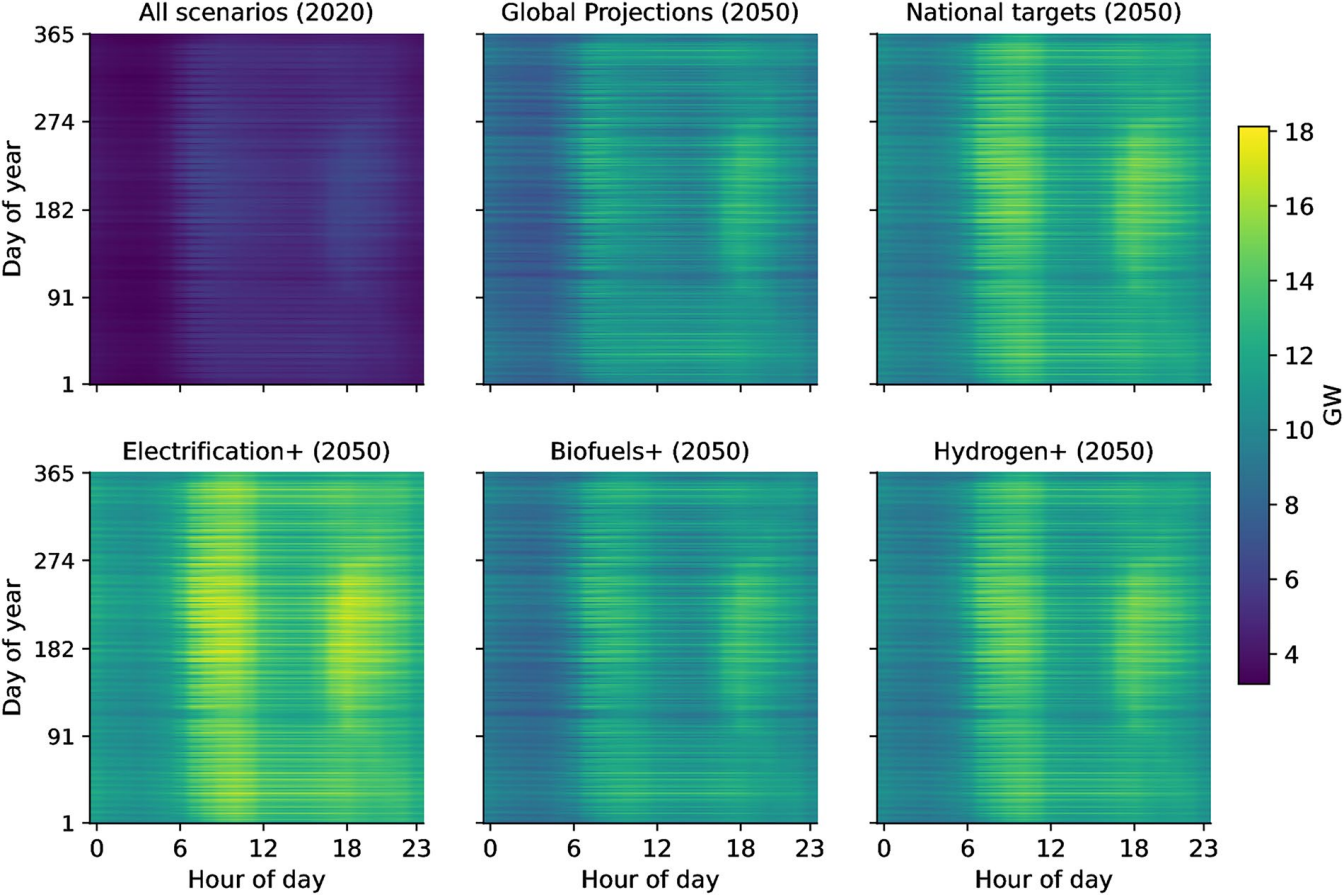
Annual hourly load carpets for 2020 and 2050 across scenarios. Each panel shows the average national hourly demand (GW) reshaped into a day-hour matrix (365 days, 24 hours). The top-left panel represents the 2020 average (identical for all scenarios), while the remaining panels show results for 2050 under each scenario. Colour scale is shared across all panels for comparability.

This section provides a brief visual summary of the dataset^29^. Figure 5 shows annual energy demand for power, heat, and transport across all scenarios, together with corresponding five-year change rates. Figure 6 displays hourly plots to show electricity demand profiles throughout the year. All values shown in the figures are taken directly from the dataset^29^. Users are encouraged to download the data and perform further analysis if required.

## Technical Validation

The dataset^29^ was validated for each sector to verify the integrity and logical consistency of the data.

### Heating and cooling

All five scenarios produced remained within ±30% of the reference values in a global study^26^, confirming that the dataset^29^ is of the same order of magnitude as values reported in the literature (see Table 12). The larger deviations observed in NT and ELEC + stem from their design: NT is constrained by national targets, particularly the mandated gas phase-out in industrial process heat by 2030, while ELEC + builds on NT with even stronger electrification assumptions, leading to a greater reduction in energy demand.

**Table 12:** Deviation of scenario heat demand from literature reference^26^ (Avg. Dev. = average deviation, Min./Max. Abs. Dev. = minimum/maximum absolute deviation).

Deviation from reference^26^	2020	2025	2030	2035	2040	2045	2050	Avg. Dev.	Min. Abs. Dev.	Max. Abs. Dev
Global Projections (GP)	−19%	−17%	−15%	−13%	−10%	−7%	−2%	−12%	2%	19%
National Targets (NT)	−19%	−21%	−24%	−26%	−26%	−23%	−20%	−23%	19%	26%
Electrification (ELEC+)	−19%	−23%	−27%	−30%	−31%	−29%	−26%	−26%	19%	31%
Biomass (BIO+)	−19%	−18%	−18%	−16%	−13%	−9%	−4%	−14%	4%	19%
Hydrogen (H₂+)	−19%	−18%	−18%	−16%	−13%	−9%	−4%	−14%	4%	19%
Average deviation	−19%	−19%	−20%	−20%	−19%	−15%	−11%	−18%	11%	20%

The heat-demand projection already incorporates temperature changes associated with climate change, following Keiner *et al*.^26^, who derive annual temperature adjustments from the IPCC AR4 B1 scenario. Long-term warming effects are therefore reflected in the space-heating trajectory. To support simple climate-sensitivity exploration, we provide two additional hourly variants for each region and sector representing a warmer and a cooler year. These profiles are generated by scaling the heating-related hourly components using HDD/CDD adjustments as guidance. The resulting variants are included in the dataset^29^ in Heat_profile_with_variants.csv (see Table 9) and serve as generic sensitivity options. Users who require alternative climate pathways may rescale the published profiles using their own HDD/CDD factors or degree-hour scalars (e.g., based on Representative Concentration Pathway (RCP) 2.6 or RCP 4.5 projections^70^).

### Transportation

**Fig. 7:**
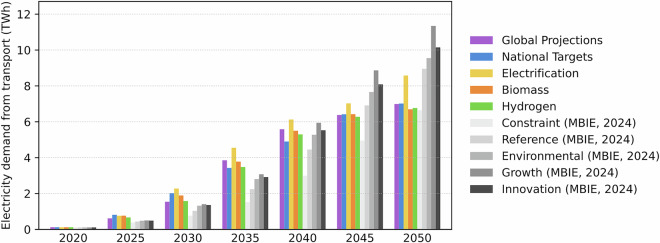
Projected electricity demand from transport: comparison with MBIE Electricity Demand and Generation Scenarios (EDGS)^71^.

Modelled transport electricity demand aligns closely with national MBIE projections^71^ through 2030, but diverges beyond 2040, remaining consistently lower (see Fig. 7). This reflects differences in scenario assumptions, including coverage of transport modes, EV uptake rates, and total travel activity. In our dataset^29^, scenarios include earlier adoption for buses and light-duty vehicles, whereas EDGS limits electrification to 76% of VKT and does not report conversion or efficiency assumptions^72^.

### Power sector

Nodal electricity demand was downloaded in trading-period (half-hourly) resolution for 2019 in monthly files^37^ and compared against the reported national totals^73^. Aggregated nodal values showed agreement with reported monthly demand, with deviations consistently below 0.2%. After validation, nodal series were checked for completeness, confirming the availability of 8,760 hourly values for all 162 nodes.

**Fig. 8:**
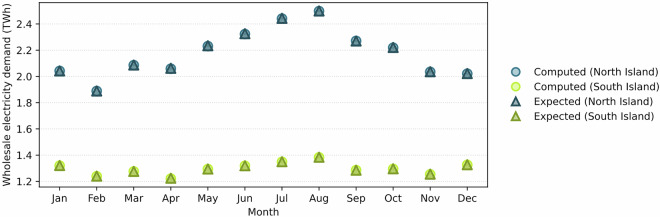
Monthly electricity demand by island (North and South): computed from validated nodal data vs official Electricity Authority totals^73^.

To further assess accuracy, monthly nodal demand was aggregated to the island level and compared with reported totals for the North and South Islands^73^. Deviations remained below 0.2% across all months (Fig. 8). This validation ensures that the nodal dataset^29^ provides a reliable basis for subsequent projection.

### Total energy demand

**Fig. 9:**
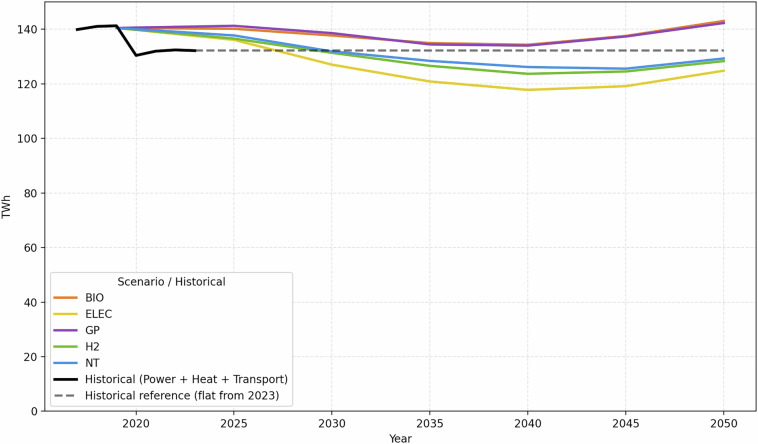
Total final energy demand (heat, transport, power): historical reference^30^ vs scenario projections.

Historical total final energy demand across New Zealand’s heat, transport, and power sectors ranged between 130–141 TWh from 2017 to 2023, with a noticeable dip in 2020 due to COVID-19 (see Fig. 9). By 2023, demand reached 132 TWh, still below the 2019 level. The scenario estimates for 2025 fall within or slightly above the recent historical range (136–141 TWh, median 138 TWh, +4% relative to 2023), suggesting appropriate scaling.

## Usage Notes

This dataset^29^ is intended to support energy system modelling, planning, and decarbonisation studies that require regionally and temporally resolved demand profiles. The hourly electricity demand series can be used directly as inputs to operation and capacity expansion models, and the annual sectoral energy totals can be incorporated into broader scenario analyses, demand projections, or optimisation workflows. Users can extract demand for specific regions, sectors, or scenarios using the region, sector, and scenario identifiers provided in the summary and hourly data files. The files are structured to enable rapid filtering, aggregation, or integration into modelling workflows. Users are encouraged to consult the Data record section for information on file formats, field definitions, and directory structure.

### Technical example: retrieving and combining data fields

The following brief example illustrates how users may access and combine fields from the workbooks (10.5281/zenodo.17191686)^29^. To obtain the hourly electricity-demand profile for Canterbury, National Targets, 2045, and confirm how to construct total electricity demand without double counting, the user can:

1. Locate the hourly profile

- Open Energy_summary.xlsx, sheet E3_Hourly_Heat.
- Filter rows by: Region = Canterbury, Scenario = NT, Year = 2045.
- The resulting row contains columns t0001 to t8760, which represent hourly demand for heat-sector electricity.

2. Repeat for other sectors if needed

- Transport hourly electricity profiles are in E3_Hourly_Transport.
- Power-sector electricity demand is in E3_Hourly_Power (this sheet reports consumption within the power sector itself and does not include heat or transport; therefore, summing all three sheets does not produce double counting).

3. Combine hourly layers

- To construct total national hourly electricity demand sum the heat, transport, and power hourly profiles for matching Scenario, Region, Year, and hour.
- No additional corrections are required to avoid double counting, as each sheet reports demand for its respective sector only.

This process applies consistently across all scenarios and years.

### Sensitivity multipliers and climate-related variants

For applications requiring simple sensitivity analysis, the dataset^29^ includes a small set of High/Low multipliers for key drivers (e.g., ±10% activity growth, ±5–10% technology efficiency variation, and alternative penetration of heat pumps for space heating). These multipliers are intended as optional, tools to support exploratory analyses and are not formal uncertainty bounds.

To support basic climate‐sensitivity exploration, two additional hourly profiles are provided in Heat_profile_with_variants.csv^29^ for each sector and region: a warmer-year and a cooler-year variant. These profiles were generated through HDD/CDD-based scaling of the baseline hourly shapes. Users may also rescale the published heating profiles using their own HDD/CDD factors or externally derived climate projections if alternative climate futures are required.

### Reproducibility resources

A small reproducibility package is also included in the Zenodo record (10.5281/zenodo.17191686)^29^. It provides a Python script to reconstruct a single sector/scenario/year from the workbooks; an environment file with pinned package versions; and a README file describing the contents and workflow. This provides a transparent example of how regional and hourly values can be combined to reproduce the national annual totals supplied in the dataset^29^.

### Additional notes

This dataset^29^ does not include carbon prices, emission caps, or carbon budgets. These are typically applied inside the energy-system model where emissions are calculated. The file Supporting_data.xlsx (tab: Carbon)^29^ includes a small non-authoritative table of New Zealand grid emission factors^40^, New Zealand Emissions Trading System auction prices^74^, and IPCC SR15 carbon-price points^75^.

## Data Availability

The full data files are archived and publicly available from the Zenodo record (10.5281/zenodo.17191686)^29^.

## Code availability

No custom code is required to reuse the dataset^29^. Processing and plotting scripts were used to reorganize and visualise the data, but these are not necessary to apply the dataset^29^. They are available from the corresponding author upon reasonable request. To support transparency and reproducibility, a minimal code bundle has also been deposited in the Zenodo record (10.5281/zenodo.17191686)^29^. The bundle includes a Python example that reconstructs a single sector/scenario/year directly from the workbooks, together with pinned package versions and a README describing the workflow. This example illustrates how hourly and regional values can be aggregated to reproduce the national annual totals provided in the dataset.
